# DNA methylation signatures in human neonatal blood following maternal antenatal corticosteroid treatment

**DOI:** 10.1038/s41398-022-01902-4

**Published:** 2022-03-31

**Authors:** Bona Kim, Aya Sasaki, Kellie Murphy, Stephen G. Matthews

**Affiliations:** 1 Department of Physiology, University of Toronto, Toronto, ON Canada; 2 Lunenfeld-Tanenbaum Research Institute, Sinai Health System, Toronto, ON Canada; 3 Department of Obstetrics & Gynecology, University of Toronto, Toronto, ON Canada

**Keywords:** Clinical genetics, Physiology, Comparative genomics

## Abstract

Antenatal corticosteroids (ACS) are used to treat women at risk of preterm birth to improve neonatal survival. Though affected children may be at long-term risk of neurobehavioural disorders, the driving mechanisms remain unknown. Animal studies have shown that ACS exposure can lead to overlapping changes in DNA methylation between the blood and the brain, identifying gene pathways for neurodevelopment, which highlights the potential to examine peripheral blood as a surrogate for inaccessible human brain tissue. We hypothesized that differential methylation will be identified in blood of term-born neonates following ACS. Mother-infant dyads that received ACS were retrospectively identified through the Ontario Birth Study at Sinai Health Complex and matched to untreated controls for maternal age, BMI, parity and foetal sex (*n* = 14/group). Genome-wide methylation differences were examined at single-nucleotide resolution in DNA extracted from dried bloodspot cards using reduced representative bisulfite sequencing approaches. 505 differentially methylated CpG sites (DMCs) were identified, wherein 231 were hypermethylated and 274 were hypomethylated. These sites were annotated to 219 genes, of which *USP48, SH3PXD2A, NTM, CAMK2N2, MAP6D1* were five of the top ten genes with known neurological function. Collectively, the set of hypermethylated genes were enriched for pathways of transcription regulation, while pathways of proteasome activity were enriched among the set of hypomethylated genes. This study is the first to identify DNA methylation changes in human neonatal blood following ACS. Understanding the epigenetic changes that occur in response to ACS will support future investigations to delineate the effects of prenatal glucocorticoid exposure on human development.

## Introduction

Antenatal corticosteroids (ACS) are synthetic glucocorticoids that are prescribed to women at risk of preterm birth to improve perinatal survival and decrease morbidity. Glucocorticoids act to accelerate foetal organ maturation to reduce the incidence of neonatal respiratory distress syndrome and intraventricular haemorrhage. Epidemiological studies have identified associations between ACS exposure and increased risk for a variety of cardiometabolic, immune and neurodevelopmental dissorders [1–3] affecting multiple organ systems. These associations have been shown to occur independent of preterm birth, a known confounder for many disorders [4, 5]. At the level of neurodevelopmental outcomes, Räikkönen *et al*. reported that term-born children exposed to ACS are at increased risk of various mental or behavioural disorders, including disorders of attention/hyperactivity, conduct, emotional and social functioning and sleep [6]. Altered regulation of the hypothalamic-pituitary-adrenal (HPA) axis has also been reported, where female children exposed to ACS demonstrated higher salivary cortisol levels than unexposed subjects in a standardized laboratory stress test (Trier Social Stress Test) [7]. The effects of altered developmental trajectories may be long-lasting, as altered HPA axis reactivity, higher-order cognitive and decision-making capacities have been observed throughout childhood and into adolescence in offspring antenatally exposed to ACS [7–9].

Despite phenotypic evidence suggesting long-term consequences of ACS treatment, little is known about the potential mechanisms involved. Given that epigenetic modifications are responsive to environmental stimuli [10, 11], modifications like DNA methylation (DNAm) which are sustained across cell divisions [12] represent a likely candidate for mediating long-lasting phenotypic changes in response to acute environmental exposures. Previous animal studies have demonstrated that ACS exposure leaves both acute [13, 14] and long-term [15] genome-wide methylation changes in the hippocampus of exposed offspring, which were associated with a hyperactive phenotype [15], thus identifying a shifted methylome as a possible mediator between ACS exposure and altered phenotypes.

Healthy brain tissue is not easily accessible in humans, thus mechanistic investigations have often relied on surrogate markers derived from peripheral tissues such as blood. The blood methylome is a valuable surrogate for the DNAm status in various regions of the brain, with high correlations (*r* = 0.86) reported in average methylation levels between blood and live brain samples resected during neurosurgery [16]. However, methylation correlations between brain and peripheral tissue can be complex, dependent on factors such as whether samples were obtained post-mortem [17], if the subjects suffered from brain disorders [18], or if comparisons were conducted across-subjects (interindividual) or within-subject [16]. As such, we have previously examined methylation correspondence in the context of ACS treatment and reported a positive correlation (r = 0.31) in methylation changes between the blood and hippocampus of female guinea pig offspring, collected simultaneously, following ACS exposure. In this analysis, ≥1000 differentially methylated sites were identified in common [19], which annotated to 134 genes that were enriched for gene pathways involved in neurodevelopment [19]. Together, these data support the use of peripheral blood as a surrogate for investigating methylation changes in the brain following ACS exposure.

In the present study, we investigated the effects of ACS on DNAm in a cohort of term-born neonates. We hypothesized that ACS exposure will alter DNA methylation events in neonatal whole blood. Differentially methylated sites will be examined to identify affected genes and enriched pathways to understand the functional networks that are targeted by ACS exposure.

## Methods

### Study participants

Study participants were retrospectively identified through the Ontario Birth Study (OBS) [20] conducted at Sinai Health Systems (REB:17-0210-E, Toronto, Canada). All subjects provided informed consent upon OBS enrolment. All term-born infants that were exposed to ACS treatment (Celestone®, Betamethasone, single course: 12 mg x 2, 24 h apart) between 24–33 weeks gestation were identified. Subjects with indication of maternal use of chronic glucocorticoids, known intrauterine growth restriction, in vitro fertilization, maternal diabetes, and known infectious diseases were excluded. Only subjects for whom OBS had collected neonatal heel prick blood spot cards were included in this study (~40% of all OBS participants). As per OBS protocol, blood spot cards were collected at the time of routine heel pricks used for clinical diagnoses (24 h post-birth), so as to not impart additional invasive procedures for research purposes. We have previously shown that methylation profiles are resilient between whole blood and dried blood spot cards [21]. Identified case subjects (*n* = 14, 8 male, 6 female) represented all of the samples available (following removal of excluded participants) from the entire OBS repository of ~3000 participants and were matched to controls for maternal age, maternal BMI, parity, and foetal sex. Descriptive characteristics are shown in Table 1. All subjects and biospecimens were assigned unique identification numbers to protect patient identity from research personnel.

**Table 1:** Descriptive characteristics of study participants.

		ACS (*n* = 14)	Control (*n* = 14)	*p*-value
Maternal Age	Mean (SD)	34.7 (3.3)	33.6 (2.8)	0.36
Parity	0	6	6	0.42
	1	4	7	
	2	3	1	
	3	1	0	
BMI	Average BMI (SD)	27.3 (5.0)	26.6 (5.0)	0.76^a^
GW at ACS	Mean (SD)	27.9 (2.4)	n/a	
Birthweight	Grams (SD)	3407.3 (619.2)	3687.3 (407.2)	0.21
Infant sex	Male	8	8	> 0.99
	Female	6	6	
Mode of Delivery	C/S	7	5	0.7
	Vaginal	7	9	

^a^*P*-value for average BMI was calculated using student *t*-test.

### DNA preparation

Genomic DNA was extracted from dried blood spot cards using the GenSolve DNA COMPLETE Kit (GenTegra, Pleasanton, CA, USA) according to the manufacturer’s instructions. Genomic DNA was quantified with Quant-iT Picogreen dsDNA assay (ThermoFisher, Waltham, MA, USA), and quality was assessed using TapeStation (Agilent, Santa Clara, CA, USA) at the Centre for Applied Genomics at the Peter Gilgan Centre for Research and Learning (Hospital for Sick Children, Toronto, Canada). Reduced Representation Bisulfite Sequencing (RRBS) libraries were prepared from 100 ng of high-quality dsDNA (DNA Integrity Number (DIN) greater than 7) using the Ovation RRBS Methyl-Seq System 1-16 (Tecan Genomics, Redwood City, CA, USA) and EpiTect Fast DNA Bisulfite kit (Qiagen, Germany) following the manufacturer’s protocols. RRBS libraries were sequenced using Illumina’s NextSeq500 platform at the Donnelly Sequencing Centre (University of Toronto, Toronto, Canada), using single-end reads of 75 bp read lengths as per manufacturer protocol. Samples were sequenced in pooled multiplexes of 10, balanced for treatment condition to minimize potential batch effects.

### Bioinformatic identification of differentially methylated sites

Sequenced reads were trimmed to remove Illumina adaptor sequences and low-quality reads with Phred quality scores <30 using *Trim Galore (*v 0.6.4*)*, (https://www.bioinformatics.babraham.ac.uk/projects/trim_galore/) which is a wrapper script around CutAdapt [22]. Reads that did not contain an MspI site signature at the 5′ end were removed using a Python script provided by NuGEN (Github: trimRRBSdiversityAdaptCustomers.py). Trimmed and filtered reads were aligned to the human genome (hg38) using *Bismark (*v0.16.0*)* [23] and *Bowtie2* (v2.3.4.3) [24]. Reads were then sorted by *Samtools (v1.9)* [25]. Aligned and sorted reads were analyzed by *MethPipe* [26, 27], to identify differentially methylated CpG sites (DMC) between ACS-treated and untreated samples at single-nucleotide resolution. Briefly, *methpipe/methcounts* obtained methylation levels at individual cytosines, which was used to perform beta-binomial regression analyses in the *methpipe/radmeth* programme under the *regression* option. Identified CpG sites were adjusted (bins 1:200:1) based on neighbouring sites using the *adjust* option in *methpipe/radmeth* to obtain a FDR-corrected list of DMCs. Analysis was limited to DMCs with at least 30x coverage with ≥5% methylation difference between ACS-treated and control subjects and false discovery rates (FDR) < 0.05. Computations were performed on the Niagara supercomputer at the SciNet HPC Consortium [28].

### Gene enrichment analysis

Differentially methylated sites were annotated to known genes to analyze their functional relevance. This was performed by referencing individual DMC coordinates on the human genome assembly (hg38) using UCSC’s genome browser (http://genome.ucsc.edu/). DMCs localized within gene bodies (genic) were annotated to the identified gene. As methylation changes in regulatory regions (promoters, enhancers, DNase-H3K4me3) present the most pronounced functional effects [29], DMCs localized within regulatory elements were also annotated to their gene targets. DNase-H3K4me3 regions refer to accessible regions of the chromatin that share biochemical signals similar to promoters, which can function as transcription factor binding sites. Regulatory elements were analyzed in two subsequent steps to identify target genes or interacting transcription factors. Target genes were identified by searching the unique GeneHancer numbers (GH-) of individual regulatory elements within the GeneCards database (genecards.org). These numbers are catalogued to find associations between the regulatory elements and target genes by calling on seven different genome-wide databases [30]. DNase-H3K4me3 regions and their binding transcription factors were identified by ENCODE Accession numbers (EH-) for candidate cis-regulatory elements (cCRE), which indicate known interacting transcription factors and the cell type in which the interaction occurs, based on ChIP-seq experiments. ENCODE Accession numbers can be searched on version 2 of SCREEN: Search Candidate cis-Regulatory Elements by ENCODE (https://screen-v2.wenglab.org/) [31, 32]. Tissue-specific expression of genes were examined on the Genotype-Tissue Expresion (GTEx) portal (https://gtexportal.org/home/) [33]. The identified genes were then used to perform an enriched biological pathways analysis to understand gene interactions using the STRING portal [34] *(*https://version11.string-db.org*)*, which sources different databases (Gene Enrichment Ontology, KEGG, Reactome) to predict interactions. Pathways were analyzed separately for genes sets of hypermethylated and hypomethylated DMCs to better understand functional implications of their interactions. Significant enrichment was defined as FDR < 0.05.

## Results

### Differentially methylated CpG sites

**Fig. 1:**
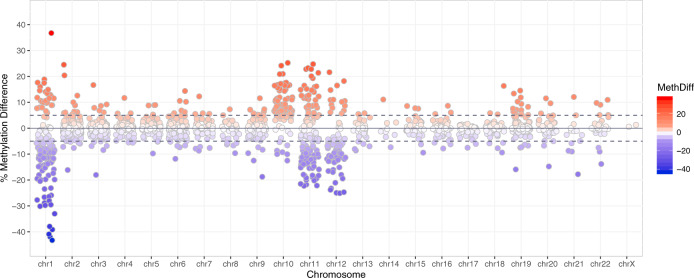
Overview of differentially methylated CpG sites (DMC) in peripheral whole blood of neonates exposed to ACS treatment. Scatterplot of individual CpG sites that were significantly differentially methylated in ACS-exposed subjects as compared to unexposed controls. Each dot represents a DMC, visualized across the genome (FDR < 0.05). Chromosomes are displayed in numerical order along the x-axis. DMCs above and below the dashed line (≥5% methylation difference, 505 sites) were included in the analysis. Red dots above the dashed line represent hypermethylated DMCS that demonstrated greater than 5% methylation difference (231 DMC (46%)). Blue dots under the dashed line represent hypomethylated DMCs that demonstrated greater than 5% methylation difference (274 DMC (54%)).

In total, 505 differentially methylated sites (DMCs) were identified in human neonatal blood following ACS treatment (≥5% methylation difference, FDR < 0.05 for *n* = 14/treatment), of which 231 sites were hypermethylated, representing 46%, and 274 were hypomethylated (54%) (Fig. 1). Full list of DMCs can be found in Supplementary Table 1.

Sites were visualized on the UCSC genome browser (hg38) to map their genomic features (genic, promoter, enhancer, DNase-H3K4me3). Not all DMCs were localized within known genomic regions. Annotated sites were localized to 44 genic regions, 37 enhancer regions, 25 promoter regions and four DNase-H3K4me3 regions. In 30 instances, regulatory elements (promoter/enhancer/DNase-H3K4me3) were observed in the same region as intragenic segments.

**Fig. 2:**
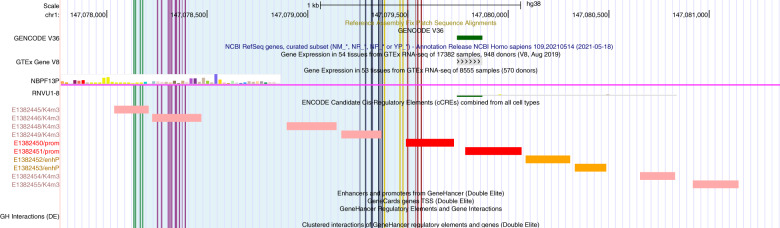
Genomic features visualized relative to transcription factor binding site EH38E1382446 that was identified in the list of top 20 DMCs. The 1432 base-pair region including 35 hypomethylated DMCs is highlighted in blue. Vertical bars indicate individual DMCs that were identified. The 15 DMCs that were identified within EH38E1382446 are highlighted in purple. 4 DMCs highlighted by green bars are mapped to DNase-H3K4me3 EH38E1382445, navy bars (7 DMCs) are mapped to DNase-H3K4me3 EH38E1382449. Yellow bars show 4 DMCs that are not annotated to any known genomic features, and red bars show 4 DMCs that are mapped to promoter E1382450. (Image obtained from UCSC genome browser for hg38, region chr1:146,956,350-147,200,349).

To identify DMCs where the greatest differences in methylation events occurred, sites were reorganized to demonstrate the top 20 DMCs (Table 2). 15 sites, all hypomethylated (−25.91 to −43.25%), were localized within one DNase-H3K4me3 region (EH38E1382446) between chr1: 147078251-147078392 (141 base pairs). Examined in context of all 505 DMCs, region EH38E1382446 was observed in proximity to two additional DNase-H3K4me3 regions (EH38E1382445, EH38E1382449) and one promoter (E1382450), which were all hypomethylated (−7.89 to −43.25%, avg −22.55%). 35 DMCs were identified in this region (Fig. 2), spanning 1432 base-pairs (chr1: 147078133-147079565).

**Table 2:** List of Top 20 differentially methylated CpG sites.

Rank	Locus	FDR	% Methylation difference^a^	Genomic feature	Identifier^b^	Associated gene
**1**	1: 147078360	2.89E−07	−43.25	TF Binding Site	EH38E1382446	
**2**	1: 147078341	1.68E−07	−41.92	TF Binding Site	EH38E1382446	
**3**	1: 147078347	2.89E−07	−40.91	TF Binding Site	EH38E1382446	
**4**	1: 147078274	8.25E−08	−39.13	TF Binding Site	EH38E1382446	
**5**	1: 147078323	1.41E−07	−37.93	TF Binding Site	EH38E1382446	
**6**	1: 21957008	2.66E−02	36.73	Promoter	GH01J021955	HSPG2, USP48, CELA3B
**7**	1: 147078315	8.25E−08	−33.02	TF Binding Site	EH38E1382446	
**8**	1: 147078303	8.25E−08	−30.14	TF Binding Site	EH38E1382446	
**9**	1: 147078373	4.10E−07	−29.77	TF Binding Site	EH38E1382446	
**10**	1: 147079539	7.33E−03	−29.54	TF Binding Site	EH38E1382446	
**11**	1: 147078348	2.89E−07	−28.86	TF Binding Site	EH38E1382446	
**12**	1: 147078392	5.00E−07	−28.02	TF Binding Site	EH38E1382446	
**13**	1: 147078324	1.41E−07	−27.71	TF Binding Site	EH38E1382446	
**14**	1: 147078342	1.68E−07	−26.62	TF Binding Site	EH38E1382446	
**15**	1: 147078361	2.89E−07	−26.40	TF Binding Site	EH38E1382446	
**16**	1: 147078251	6.51E−08	−25.91	TF Binding Site	EH38E1382446	
**17**	10: 103661550	2.92E−02	25.23	Gene		SH3PXD2A
**18**	12: 110230095	5.85E−03	−25.08	n/a		
**19**	12: 110230084	5.85E−03	−24.96	n/a		
**20**	11: 132093969	6.03E−03	24.76	Gene / Promoter	EH38E1583631	NTM

Top DMCs ranked by significance (lowest to highest FDR-values). Negative methylation difference indicates hypomethylation in the ACS-treated group compared to untreated controls. ^a^% methylation difference is calculated as case–control. Positive methylation difference indicates hypermethylation in the ACS group compared to untreated controls. ^b^IDs beginning with EH- refer to ENCODE cCRE Accession Numbers, and IDs starting with GH refer to GeneHancer IDs.

### Gene set enrichment analysis

DMCs were annotated to known genes and/or regulatory features to understand the functional implications of the altered methylome. DMCs in regulatory regions (promoter, enhancer, DNase-H3K4me3) were annotated to their target genes, as described in methods above. All DNase-H3K4me3 regions identified were functional transcription factor binding sites (TFBS) as evidenced by ChIP-Seq experiments, reported on SCREEN (https://screen-v2.wenglab.org/).

**Fig. 3:**
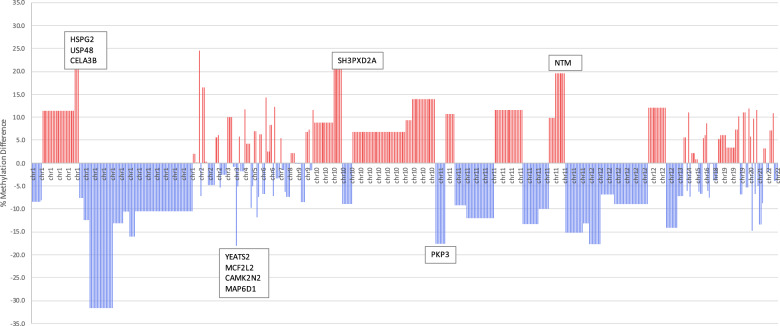
Differentially methylated sites represented to display average methylation differences as a function of genomic regions. Bar graph of individual CpG sites that were differentially methylated in ACS-exposed subjects as compared to unexposed controls (DMC). Each bar represents a DMC, visualized across the genome displayed along the x-axis. Average methylation difference % is represented along the y-axis such that adjacent bars of same height represent DMCs that are located within the same region. Labelled are the top ten differentially methylated genes.

In total, 219 genes were identified (Supplementary Table 2). 74 genes were annotated to hypermethylated DMCs, and 100 genes were annotated to hypomethylated sites. 45 genes were annotated to regions where methylation changes occurred in both directions. Of the top ten differentially methylated genes (*HSPG2, USP48, CELA3B, SH3PXD2A, NTM, YEATS2, MCF2L2, CAMK2N2, MAP6D1, PKP3)* (Fig. 3), four genes (*HSPG2, USP48, CELA3B, NTM*) contained glucocorticoid response elements (GRE) within their promoter regions, and five genes (*USP48, SH3PXD2A, NTM, CAMK2N2, MAP6D1*) are known to be highly expressed in the brain and have neurological roles (Table 3).

**Table 3:** List of Top 10 differentially methylated genes.

Gene	Locus	Meth Diff %	Avg Meth %	Gene description
HSPG2	chr1: 21956970-21957010	14.53~17.63	22.96	Heparan Sulfate Proteoglycan 2
USP48	chr1: 21956970-21957011	14.53~17.64	22.96	Ubiquitin Specific Peptidase 48
CELA3B	chr1: 21956970-21957012	14.53~17.65	22.96	Chymotrypsin Like Elastase 3b (Pancreatic endopeptidase E)
SH3PXD2A	chr10:103661518-103661558	16.66~25.23	20.87	SH3 and PX Domains 2A (tyrosine Kinase substrate w/ five SH3 domains)
NTM	chr11:132093921-132094039	12.59~24.76	19.64	Neurotrimin
YEATS2	chr3:183776202	−18.04	−18.04	YEATS Domain Containing 2
MCF2L2	chr3:183776203	−18.04	−18.04	MCF.2 Cell Line Derived Transforming Sequence-Like 2
CAMK2N2	chr3:183776204	−18.04	−18.04	Calcium/Calmodulin Dependent Protein Kinase II Inhibitor 2
MAP6D1	chr3:183776205	−18.04	−18.04	MAP6 Domain-Containing Protein 1
PKP3	chr11:400486-403008	−8.58~−21.77	*−*17.60	Plakophilin 3

Top differentially methylated genes ranked by decreasing average methylation difference. Genes are identified with their chromosomal location (locus), the range of methylation difference observed at individual DMCs annotated to the gene, and the average methylation difference of the DMCs. Bolded are genes with known neurological roles

Gene set enrichment analysis of the identified gene set was performed using STRING (https://version11.string-db.org/), to identify shared biological functions. The entire set of genes observed together was significantly enriched (*P* = 0.000855) for transcription regulation (Reactome, FDR = 0.02). This analysis was repeated to assess the pathways independently enriched by hypermethylated and hypomethylated genes. Hypermethylated genes were similarly enriched (*P* = 0.0121) in pathways of transcription regulation (GO Molecular Function, FDR = 0.0063) and generic transcription (Reactome, FDR = 0.00036). Hypomethylated genes were enriched (P = 1.07e−05) for pathways of proteasome activity (GO Molecular Function, FDR = 0.01; KEGG, FDR = 0.03; UniProt, FDR = 0.03).

## Discussion

In this study, we have identified, for the first time, that ACS treatment results in profound changes in DNA methylation in human neonatal blood. A number of the genes that we have identified were also differentially methylated in blood and hippocampus in guinea pigs following ACS treatment, and these enriched for pathways of neurodevelopment [19]. The greatest methylation differences were observed in a 1432 base-pair region where 35 DMCs were hypomethylated in the ACS exposed group and annotated to three DNase-H3K4me3 regions, which functioned as transcription factor binding sites (EH38E1382445, EH38E1382446, EH38E1382449) and one promoter (EH382450). ChIP-seq experiments indicate that six transcription repressors bind to these TFBS (*EZH2, SIN3A, SUZ12, CHD1, USF1, KDM4A*) in various cell lines, including B-lymphocytes (GM12878, ENCSR000ARD), neural cells (H1 differentiated in vitro, ENCSR511CUH) and neural progenitor cells (H9 differentiated in vitro, ENCSR069DPL) [35], indicating potential implications for transcription of target genes in both blood and brain tissue. Target genes of these TFBS regions and promoter EH382450 include *NBPF12, CHD1L, FMO5* [36] and *PRKAB2* (by proximity), of which *NBPF12* (neuroblastoma breakpoint family member 12) and *CHD1L* (chromodomain-helicase-DNA-binding protein 1-like) are expressed in brain and whole blood, as demonstrated on GTEx.

*CHD1L* is a glucocorticoid-responsive gene [37, 38] involved in chromatin remodelling that promotes proliferation and pluripotency [39, 40]. The role of *CHD1L* has been thoroughly described in cancer literature to drive tumour proliferation lead to poor cancer prognosis [41–43] possibly by regulating immune factors in regulating apoptosis such as CD8+ T-cell populations and IL-6 levels [44]. *NFBP12* is also a gene implicated in cell proliferation, primarily involved in neural stem cell proliferation during cortical neurogenesis, leading to altered brain size [45]. Reduced proliferation in numerous cell types [46–48], including neural stem cells [48, 49] has been observed in response to high levels of glucocorticoid exposure, leading to reduced neuronal proliferation and brain volume and weight in rodents [48] and non-human primates [50]. Reduced hippocampal volume was maintained at 20 months of age [51] in non-human primates prenatally exposed to dexamethasone. In humans, cortical thinning of the rostral anterior cingulate cortex has been observed in 6-10 year-old children treated with ACS (and born at term) [52], suggesting that prenatal glucocorticoid exposure can result in long-term reduction of cellular proliferation in various brain regions. It is possible that hypomethylation of regulatory elements that mediate expression of *NFBP12* and *CHD1L* persist throughout development to affect neuronal proliferation. The association of these genes to neurodevelopmental disorders that have been observed in ACS-exposed children such as cognitive disability and attention-deficit-hyperactive-disorder [53–55] makes them strong targets for further investigation.

Synaptic development, including synaptogenesis and synaptic plasticity, is another key component of normal brain development that can lead to altered outcomes in learning and memory [56, 57]. Glucocorticoid-mediated alterations in synaptic development have been shown in both in vitro and in vivo models [58–60]. As ACS is often administered during a window of high synaptic development [61], it is important to examine the effects of ACS on synaptogenic events.

*NTM* (Neurotrimin) is a member of a family of neural cell adhesion molecules that was one of the top ten differentially methylated genes in this study. Along with *CDH4* (cadherin 4), which was also identified to be differentially methylated in both the current study and the previously conducted guinea pig study [19], these genes are known to be implicated in growth cone migration, axon guidance, and synapse formation and stabilization throughout development and into adulthood [62, 63]. However, altered regulation of *NTM* can lead to synaptic overgrowth and dysfunction, resulting in unexpected phenotypes in affected individuals. *CAMK2N2* (Calcium/calmodulin dependent protein kinase II inhibitor 2), is another gene implicated in the regulation of synaptic plasticity that was strongly differentially methylated in response to ACS exposure. *CAMK2N2* encodes for a gene which functions to inhibit the phosphorylation of AMPA receptors in the post-synaptic neuron by CamKII [64]. It was demonstrated in the male guinea pig hippocampus that prenatal exposure to betamethsone resulted in reduced levels of phosphorylated-CamKII (the active form), while exposed female offspring were resistant to reduced LTP events triggered by secondary stimulus by cortisol [65], suggesting that ACS may influence various pathways which regulate synaptic plasticity.

*USP48* (ubiquitin specific peptidase 48) is of particular interest for its role as a deubiquitinating enzyme [66]. *USP48* has been shown to be enriched in the post-synaptic dendrites of cortical and hippocampal neurons [67], as well as in lymphocytes (EBV-transformed lymphocytes) as demonstrated on the GTEx portal, while various members of the USP family have also been shown to be differentially methylated in the blood and hippocampus of guinea pigs following ACS exposure [19]. Protein ubiquitination is a post-translational modification that tags proteins for downstream interaction with the proteasome complex which can lead to protein degradation, endocytosis, or intracellular trafficking [68, 69]. Deubiquitinating enzymes such as *USP48* remove ubiquitin from tagged proteins, thus preventing their degradation. The interaction between glucocorticoid exposure and the ubiquitin-proteasome system (UPS) has been thoroughly described [70, 71], highlighting an increased expression of ubiquitin following glucocorticoid exposure [72–74]. Following a prenatal exposure such as ACS, the methylation changes in genes that regulate the UPS may have implications for differential programming of cell function across development. In immune cells, for example, protein (de)ubiquitination can regulate antigen degradation and presentation via specialized proteasome complexes called immunoproteasomes [75, 76]. Studies to delineate the involvement of the ubiquitin-proteasome system in relationship between glucocorticoid exposure and synaptic function will also be of particular interest. In a recent 2018 publication, Choi *et al*. reported that glucocorticoid exposure led to reduced intracellular transport of AMPAR to synaptic boutons due to ubiquitin-mediated destabilization of microtubules, which resulted in memory impairment in exposed animals [77]. In our study, *MAP6D1* (MAP6 domain-containing protein 1) which encodes for a protein involved in stabilization of neuronal microtubules [78] was differentially methylated. The causal role of the *MAP6* family of genes in neuronal microtubule stability and synaptic function was established in *MAP6-KO* mice that exhibited hypo-glutamatergy in hippocampal neurons [79], vesicle depletion in synaptic densities, and impaired LTP and LTD events, which were associated with behavioural differences such as hyperactivity, increased anxiety-like behaviours, and reduced social investigation [80]. Together, differential methylation of these genes and the enrichment of the proteasomal pathway indicate possible alterations to synaptic function in response to ACS, possibly mediated by the ubiquitin-proteasome system. Future examinations of this pathway and the genetic and protein moieties involved will provide a greater understanding of the role that ACS elicits on mediating postnatal synaptic function.

A strength of this study was the ability to acquire early samples from neonates 24 hours post birth. The methylation biomarkers identified here present a very early representation of ACS exposure, which may underlie the altered neurodevelopmental trajectories that have been identified in children exposed to ACS. The early time point also ensures that the methylation markers we identified are present prior to dynamic influences of the postnatal environment to the DNA methylome [81]. While previous studies examining early time points have used cord blood for epigenetic investigation, the distinct methylation profile of nucleated red blood cells (nRBC) in cord blood [82] may significantly influence epigenetic findings [83]. The peripheral blood samples used in this study represent a time point with significantly reduced levels of circulating nRBCs [84], enabling unskewed analysis of methylation data. The relatively small sample size of this study does represent a limitation. However, the availability of these biospecimens, in a study that has recruited over 3000 women (OBS), remain limited. A larger sample size would have provided more power to perform multiple levels of analysis and examine factors such as sex-specific effects. It is also important to note, that many of the children of the OBS cohort are being followed into early childhood, and in future studies we will be positioned to examine the longer-term impact of ACS exposure on methylation patterns.

Our current ongoing studies examining targeted brain regions in guinea pigs following ACS exposure will delineate changes to not only DNA methylation, but also gene and protein expression to further elucidate the potential molecular pathways which may drive altered neurobehavioural outcomes.

This study has identified a DNA methylation signature following ACS exposure in term-born human neonatal blood. As many of the genes and pathways identified have been previously shown to be differentially methylated in both the blood and brain of juvenile guinea pigs exposed to ACS, the changes in the methylome that we have identified will enhance understanding of the biological events that occur in response to adverse exposure to prenatal glucocorticoids such as ACS or maternal stress during pregnancy. The peripheral biomarkers presented in this study may help to identify individuals who are most at risk of developing altered phenotypes and enable future studies to design targeted intervention strategies and therapies to prevent or ameliorate the effects following prenatal adversity.

## Supplementary information

Supplementary Table 1

Supplementary Table 2

## Data Availability

The datasets generated are available from the corresponding author on reasonable request and in accordance with the OBS guidelines.

## References

[1] Dean F, Yu C, Lingas RI, Matthews SG. Prenatal glucocorticoid modifies hypothalamo-pituitary-adrenal regulation in prepubertal guinea pigs. Neuroendocrinology. 2001; 10.1159/000054636. 10.1159/000054636 11307038

[2] Sparrow S, Manning JR, Cartier J, Anblagan D, Bastin ME, Piyasena C, et al. Epigenomic profiling of preterm infants reveals DNA methylation differences at sites associated with neural function. Transl Psychiatry. 2016; 10.1038/tp.2015.210. 10.1038/tp.2015.210 PMC5068883 26784970

[3] Moisiadis VG, Constantinof A, Kostaki A, Szyf M, Matthews SG. Prenatal Glucocorticoid exposure modifies endocrine function and behaviour for 3 generations following maternal and paternal transmission. Sci Rep. 2017;7: 10.1038/s41598-017-11635-w. 10.1038/s41598-017-11635-w PMC5603559 28924262

[4] Luu TM, Katz SL, Leeson P, Thébaud B, Nuyt AM. Preterm birth: risk factor for early-onset chronic diseases. CMAJ. 2016;188: 10.1503/cmaj.150450. 10.1503/cmaj.150450 PMC4938684 26644500

[5] Heuvelman H, Abel K, Wicks S, Gardner R, Johnstone E, Lee B, et al. Gestational age at birth and risk of intellectual disability without a common genetic cause. Eur J Epidemiol. 2018;33: 10.1007/s10654-017-0340-1. 10.1007/s10654-017-0340-1 PMC6061122 29214412

[6] Räikkönen K, Gissler M, Kajantie E, Associations between maternal antenatal corticosteroid treatment and mental and behavioral disorders in children. JAMA–J Am Med Assoc*.* 2020; 10.1001/jama.2020.3937. 10.1001/jama.2020.3937 PMC7237984 32427304

[7] Alexander N, Rosenlöcher F, Stalder T, Linke J, Distler W, Morgner J, et al. Impact of antenatal synthetic glucocorticoid exposure on endocrine stress reactivity in term-born children. J Clin Endocrinol Metab. 2012; 10.1210/jc.2012-1970. 10.1210/jc.2012-1970 22869608

[8] Ilg L, Kirschbaum C, Li SC, Rosenlöcher F, Miller R, Alexander N. Persistent effects of antenatal synthetic glucocorticoids on endocrine stress reactivity from childhood to adolescence. J Clin Endocrinol Metab*.* 2018; 10.1210/jc.2018-01566. 10.1210/jc.2018-01566 30285119

[9] Ilg L, Klados M, Alexander N, Kirschbaum C, Li SC. Long-term impacts of prenatal synthetic glucocorticoids exposure on functional brain correlates of cognitive monitoring in adolescence. Sci Rep. 2018; 10.1038/s41598-018-26067-3. 10.1038/s41598-018-26067-3 PMC5955898 29769646

[10] Borodinova AA, Balaban PM. Epigenetic regulation as a basis for long-term changes in the nervous system: in search of specificity mechanisms. Biochemistry. 2020;85: 10.1134/S0006297920090023. 10.1134/S0006297920090023 33050846

[11] Weinhold B. Epigenetics: the science of change. Environ Health Perspect*.* 2006;114: 10.1289/ehp.114-a160. 10.1289/ehp.114-a160 PMC1392256 16507447

[12] Vandiver AR, Idrizi A, Rizzardi L, Feinberg AP, Hansen KD. DNA methylation is stable during replication and cell cycle arrest. Sci Rep*.* 2015;5: 10.1038/srep17911. 10.1038/srep17911 PMC4673417 26648411

[13] Crudo A, Suderman M, Moisiadis VG, Petropoulos S, Kostaki A, Hallett M (2013). Glucocorticoid programming of the fetal male hippocampal epigenome. Endocrinology.

[14] Crudo A, Petropoulos S, Moisiadis VG, Iqbal M, Kostaki A, Machnes Z (2012). Prenatal synthetic glucocorticoid treatment changes DNA methylation states in male organ systems: multigenerational effects. Endocrinology.

[15] Constantinof A, Moisiadis VG, Kostaki A, Szyf M, Matthews SG. Antenatal glucocorticoid exposure results in sex-specific and transgenerational changes in prefrontal cortex gene transcription that relate to behavioural outcomes. Sci Rep*.* 2019; 10.1038/s41598-018-37088-3. 10.1038/s41598-018-37088-3 PMC6346022 30679753

[16] Braun PR, Han S, Hing B, Nagahama Y, Gaul LN, Heinzman JT, et al. Genome-wide DNA methylation comparison between live human brain and peripheral tissues within individuals. Transl Psychiatry. 2019; 9: 10.1038/s41398-019-0376-y. 10.1038/s41398-019-0376-y PMC6355837 30705257

[17] Sjöholm LK, Ransome Y, Ekström TJ, Karlsson O, Evaluation of post-mortem effects on global brain DNA methylation and hydroxymethylation. Basic Clin Pharmacol Toxicol. 2018;122: 10.1111/bcpt.12875. 10.1111/bcpt.12875 PMC5991080 28834189

[18] Walton E, Hass J, Liu J, Roffman JL, Bernardoni F, Roessner V, et al. Correspondence of DNA methylation between blood and brain tissue and its application to schizophrenia research. Schizophr Bull*.* 2016; 42: 10.1093/schbul/sbv074. 10.1093/schbul/sbv074 PMC4753587 26056378

[19] Sasaki A, Eng ME, Lee AH, Kostaki A, Matthews SG, DNA methylome signatures of prenatal exposure to synthetic glucocorticoids in hippocampus and peripheral whole blood of female guinea pigs in early life. Transl Psychiatry. 2021;11: 10.1038/s41398-020-01186-6. 10.1038/s41398-020-01186-6 PMC7813870 33462183

[20] Anderson LN, Knight JA, Hung RJ, Hewko SL, Seeto RA, Martin MJ, et al. The Ontario birth study: a prospective pregnancy cohort study integrating perinatal research into clinical care. Paediatr Perinatal Epidemiol. 2018; 32: 10.1111/ppe.12473. 10.1111/ppe.12473 29750375

[21] Sasaki A, Kim B, Murphy KE, Matthews SG, Impact of ex vivo sample handling on DNA methylation profiles in human cord blood and neonatal dried blood spots. Front Genet. 2020; 11: 10.3389/fgene.2020.00224. 10.3389/fgene.2020.00224 PMC7106936 32265984

[22] Martin M, Cutadapt removes adapter sequences from high-throughput sequencing reads. EMBnet.journal 2011; 17: 10.14806/ej.17.1.200.

[23] Krueger F, Andrews SR, Bismark: a flexible aligner and methylation caller for Bisulfite-Seq applications. Bioinformatics, 2011; 27: 10.1093/bioinformatics/btr167. 10.1093/bioinformatics/btr167 PMC3102221 21493656

[24] Langmead B, Salzberg SL, Fast gapped-read alignment with Bowtie 2. Nature Methods. Nature Publishing Group 2012. 10.1038/nmeth.1923 PMC3322381 22388286

[25] Li H, Handsaker B, Wysoker A, Fennell T, Ruan J, Homer N, et al. The Sequence Alignment/Map format and SAMtools. Bioinformatics. 2009; 25: 10.1093/bioinformatics/btp352. 10.1093/bioinformatics/btp352 PMC2723002 19505943

[26] Dolzhenko E, Smith AD, Using beta-binomial regression for high-precision differential methylation analysis in multifactor whole-genome bisulfite sequencing experiments. BMC Bioinform*.* 2014; 15: 10.1186/1471-2105-15-215. 10.1186/1471-2105-15-215 PMC4230021 24962134

[27] Song Q, Decato B, Hong EE, Zhou M, Fang F, Qu J, et al. A reference methylome database and analysis pipeline to facilitate integrative and comparative epigenomics. PLoS ONE 2013; 10.1371/journal.pone.0081148. 10.1371/journal.pone.0081148 PMC3855694 24324667

[28] Loken C, Gruner D, Groer L, Peltier R, Bunn N, Craig M, et al. SciNet: lessons learned from building a power-efficient top-20 system and data centre. In: J Phys: Conf Ser. 2010; 10.1088/1742-6596/256/1/012026.

[29] Kurdyukov S, Bullock M. DNA methylation analysis: choosing the right method. Biology. 2016; 5: 10.3390/biology5010003. 10.3390/biology5010003 PMC4810160 26751487

[30] Fishilevich S, Nudel R, Rappaport N, Hadar R, Plaschkes I, Stein TI, et al. GeneHancer: genome-wide integration of enhancers and target genes in GeneCards. Database 2017; 2017: 10.1093/database/bax028. 10.1093/database/bax028 PMC5467550 28605766

[31] Davis CA, Hitz BC, Sloan CA, Chan ET, Davidson JM, Gabdank I, et al. The Encyclopedia of DNA elements (ENCODE): data portal update. Nucleic Acids Res. 2018; 46: 10.1093/nar/gkx1081. 10.1093/nar/gkx1081 PMC5753278 29126249

[32] Abascal F, Acosta R, Addleman NJ, Adrian J, Afzal V, Aken B, et al. Expanded encyclopaedias of DNA elements in the human and mouse genomes. Nature 2020; 583: 10.1038/s41586-020-2493-4. 10.1038/s41586-020-2493-4 PMC7410828 32728249

[33] Lonsdale J, Thomas J, Salvatore M, Phillips R, Lo E, Shad S, et al. The Genotype-Tissue Expression (GTEx) project. Nat Genet. 2013; 45: 10.1038/ng.2653. 10.1038/ng.2653 PMC4010069 23715323

[34] Szklarczyk D, Gable AL, Lyon D, Junge A, Wyder S, Huerta-Cepas J, et al. STRING v11: Protein-protein association networks with increased coverage, supporting functional discovery in genome-wide experimental datasets. Nucleic Acids Res. 2019; 47: 10.1093/nar/gky1131. 10.1093/nar/gky1131 PMC6323986 30476243

[35] Wang J, Zhuang J, Iyer S, Lin XY, Greven MC, Kim BH, et al. Factorbook.org: a Wiki-based database for transcription factor-binding data generated by the ENCODE consortium. Nucleic Acids Res. 2013; 41: 10.1093/nar/gks1221. 10.1093/nar/gks1221 PMC3531197 23203885

[36] Aguet F, Brown AA, Castel SE, Davis JR, He Y, Jo B, et al. Genetic effects on gene expression across human tissues. Nature 2017; 550: 10.1038/nature24277. 10.1038/nature24277 PMC5776756 29022597

[37] Roqueta-Rivera M, Esquejo RM, Phelan PE, Sandor K, Daniel B, Foufelle F, et al. SETDB2 links glucocorticoid to lipid metabolism through Insig2a regulation. Cell Metab*.* 2016; 24: 10.1016/j.cmet.2016.07.025. 10.1016/j.cmet.2016.07.025 PMC5023502 27568546

[38] Lee BH, Stallcup MR, Glucocorticoid receptor binding to chromatin is selectively controlled by the coregulator Hic-5 and chromatin remodeling enzymes. J Biol Chem*.* 2017; 292: 10.1074/jbc.M117.782607. 10.1074/jbc.M117.782607 PMC5454112 28381557

[39] Gaspar-Maia A, Alajem A, Polesso F, Sridharan R, Mason MJ, Heidersbach A, et al. Chd1 regulates open chromatin and pluripotency of embryonic stem cells. Nature 2009; 460: 10.1038/nature08212. 10.1038/nature08212 PMC3891576 19587682

[40] Bulut-Karslioglu A, Jin H, Kim YK, Cho B, Guzman-Ayala M, Williamson AJK, et al. Chd1 protects genome integrity at promoters to sustain hypertranscription in embryonic stem cells. Nat Commun*.* 2021; 12: 10.1038/s41467-021-25088-3. 10.1038/s41467-021-25088-3 PMC8357957 34381042

[41] Li S, Chai Y, Ding Y, Yuan T, Wu C, Huang C, CHD1L is associated with poor survival and promotes the proliferation and metastasis of intrahepatic cholangiocarcinoma. Oncol Rep. 2019; 42: 10.3892/or.2019.7174. 10.3892/or.2019.7174 PMC6610041 31173252

[42] Liu C, Fu X, Zhong Z, Zhang J, Mou H, Wu Q, et al. CHD1L expression increases tumor progression and acts as a predictive biomarker for poor prognosis in pancreatic cancer. Digest. Dis Sci. 2017; 62: 10.1007/s10620-017-4641-8. 10.1007/s10620-017-4641-8 28646284

[43] Kang YY, Li JJ, Sun JX, Wei JX, Ding C, Shi CL et al. Genome-wide scanning for CHD1L gene in papillary thyroid carcinoma complicated with type 2 diabetes mellitus. Clin Transl Oncol. 2021; 10.1007/s12094-021-02656-z. 10.1007/s12094-021-02656-z 34245428

[44] Zhao D, Cai L, Lu X, Liang X, Li J, Chen P, et al. Chromatin regulator chd1 remodels the immunosuppressive tumor microenvironment in pten-deficient prostate cancer. Cancer Discov. 2020; 10: 10.1158/2159-8290.CD-19-1352. 10.1158/2159-8290.CD-19-1352 PMC7483306 32385075

[45] Keeney JG, Davis JM, Siegenthaler J, Post MD, Nielsen BS, Hopkins WD, et al. DUF1220 protein domains drive proliferation in human neural stem cells and are associated with increased cortical volume in anthropoid primates. Brain Struct Funct. 2015; 220: 10.1007/s00429-014-0814-9. 10.1007/s00429-014-0814-9 PMC4722867 24957859

[46] Xu MJ, Fang GE, Liu YJ, Song LN. Effects of glucocorticoid on proliferation, differentiation, and glucocorticoid receptor expression in human ovarian carcinoma cell line 3AO. Acta Pharmacol Sin. 2002; 23. 12230951

[47] Kanagawa T, Tomimatsu T, Hayashi S, Shioji M, Fukuda H, Shimoya K, et al. The effects of repeated corticosteroid administration on the neurogenesis in the neonatal rat. Am J Obstet Gynecol. 2006; 194: 10.1016/j.ajog.2005.06.015. 10.1016/j.ajog.2005.06.015 16389037

[48] Noorlander CW, Tijsseling D, Hessel EVS, De Vries WB, Derks JB, Visser GHA, et al. Antenatal glucocorticoid treatment affects hippocampal development in mice. PLoS ONE*.* 2014; 9: 10.1371/journal.pone.0085671. 10.1371/journal.pone.0085671 PMC3899077 24465645

[49] Provençal N, Arloth J, Cattaneo A, Anacker C, Cattane N, Wiechmann T, et al. Glucocorticoid exposure during hippocampal neurogenesis primes future stress response by inducing changes in DNA methylation. Proc Natl Acad Sci USA 2020; 117: 10.1073/pnas.1820842116. 10.1073/pnas.1820842116 PMC7519233 31399550

[50] Coe CL, Lubach GR. Developmental consequences of antenatal dexamethasone treatment in nonhuman primates. In: Neurosci Biobehav Rev. 2005; 10.1016/j.neubiorev.2004.10.003. 10.1016/j.neubiorev.2004.10.003 15811495

[51] Uno H, Lohmiller L, Thieme C, Kemnitz JW, Engle MJ, Roecker EB, et al. Brain damage induced by prenatal exposure to dexamethasone in fetal rhesus macaques. I. Hippocampus. Dev Brain Res*.* 1990; 53: 10.1016/0165-3806(90)90002-G. 10.1016/0165-3806(90)90002-g 2357788

[52] Davis EP, Sandman CA, Buss C, Wing DA, Head K F, et al. Glucocorticoid exposure is associated with preadolescent brain development. Biol Psychiatry. 2013; 74: 10.1016/j.biopsych.2013.03.009. 10.1016/j.biopsych.2013.03.009 PMC3985475 23611262

[53] Ceylan AC, Sahin I, Erdem HB, Kayhan G, Simsek-Kiper PO, Utine GE, et al. An eight-case 1q21 region series: novel aberrations and clinical variability with new features. Intellect Disabil Res*.* 2019; 63: 10.1111/jir.12592. 10.1111/jir.12592 30773728

[54] Qi X, Wang S, Zhang L, Liu L, Wen Y, Ma M, et al. An integrative analysis of transcriptome-wide association study and mRNA expression profile identified candidate genes for attention-deficit/hyperactivity disorder. Psychiatry Res. 2019; 282: 10.1016/j.psychres.2019.112639. 10.1016/j.psychres.2019.112639 31685286

[55] Kim DS, Burt AA, Ranchalis JE, Wilmot B, Smith JD, Patterson KE, et al. Sequencing of sporadic Attention-Deficit Hyperactivity Disorder (ADHD) identifies novel and potentially pathogenic de novo variants and excludes overlap with genes associated with autism spectrum disorder. Am J Med Genet Part B: Neuropsychiatr Genet*.* 2017; 174: 10.1002/ajmg.b.32527. 10.1002/ajmg.b.32527 PMC5467442 28332277

[56] Ehlers MD Activity level controls postsynaptic composition and signaling via the ubiquitin-proteasome system. Nat Neurosci. 2003; 6: 10.1038/nn1013. 10.1038/nn1013 12577062

[57] Mabb AM, Ehlers MD, Ubiquitination in postsynaptic function and plasticity. Ann Rev Cell Dev Biol. 2010; 26: 10.1146/annurev-cellbio-100109-104129. 10.1146/annurev-cellbio-100109-104129 PMC3163670 20604708

[58] Scheff SW, Dekosky ST, Glucocorticoid suppression of lesion-induced synaptogenesis: effect of temporal manipulation of steroid treatment. Exp Neurol. 1989; 105: 10.1016/0014-4886(89)90128-3. 10.1016/0014-4886(89)90128-3 2767199

[59] Myers B, McKlveen JM, Herman JP. Glucocorticoid actions on synapses, circuits, and behavior: implications for the energetics of stress. Front Neuroendocrinol. 2014; 35: 10.1016/j.yfrne.2013.12.003. 10.1016/j.yfrne.2013.12.003 PMC4422101 24361584

[60] Liston C, Gan WB. Glucocorticoids are critical regulators of dendritic spine development and plasticity in vivo. Proc National Acad Sci USA 2011; 108: 10.1073/pnas.1110444108. 10.1073/pnas.1110444108 PMC3179117 21911374

[61] Carson R, Monaghan-nichols AP, Defranco DB, Rudine AC (2016). Effects of antenatal glucocorticoids on the developing brain. Steroids.

[62] Singh K, Lilleväli K, Gilbert SF, Bregin A, Narvik J, Jayaram M, et al. The combined impact of IgLON family proteins Lsamp and Neurotrimin on developing neurons and behavioral profiles in mouse. Brain Res Bull*.* 2018; 140: 10.1016/j.brainresbull.2018.03.013. 10.1016/j.brainresbull.2018.03.013 29605488

[63] McNamee CJ, Reed JE, Howard MR, Lodge AP, Moss DJ. Promotion of neuronal cell adhesion by members of the IgLON family occurs in the absence of either support or modification of neurite outgrowth. J Neurochem. 2002; 80: 10.1046/j.0022-3042.2002.00798.x. 10.1046/j.0022-3042.2002.00798.x 11953444

[64] Astudillo D, Karmelic D, Casas BS, Otmakhov N, Palma V, Sanhueza M. CaMKII inhibitor 1 (CaMK2N1) mRNA is upregulated following LTP induction in hippocampal slices. Synapse 2020; 74: 10.1002/syn.22158. 10.1002/syn.22158 PMC8108577 32320502

[65] Setiawan E, Jackson MF, Macdonald JF, Matthews SG. Effects of repeated prenatal glucocorticoid exposure on long-term potentiation in the juvenile guinea-pig hippocampus. J Physiol*.* 2007; 10.1113/jphysiol.2006.127381. 10.1113/jphysiol.2006.127381 PMC2170854 17412773

[66] Kowalski JR, Juo P. The role of deubiquitinating enzymes in synaptic function and nervous system diseases. Neural Plast. 2012; 2012: 10.1155/2012/892749. 10.1155/2012/892749 PMC3536295 23316392

[67] Tian QB, Okano A, Nakayama K, Miyazawa S, Endo S, Suzuki T. A novel ubiquitin-specific protease, synUSP, is localized at the post-synaptic density and post-synaptic lipid raft. J Neurochem*.* 2003; 87: 10.1046/j.1471-4159.2003.02024.x. 10.1046/j.1471-4159.2003.02024.x 14535949

[68] Hershko A, Ciechanover A. The ubiquitin system. Ann Rev Biochem. 1998; 67: 10.1146/annurev.biochem.67.1.425. 10.1146/annurev.biochem.67.1.425 9759494

[69] Nath D, Shadan S The ubiquitin system. Nature. 2009; 458: 10.1038/458421a. 10.1038/458421a 19325620

[70] Conway-Campbell BL, McKenna MA, Wiles CC, Atkinson HC, De Kloet ER, Lightman SL. Proteasome-dependent down-regulation of activated nuclear hippocampal glucocorticoid receptors determines dynamic responses to corticosterone. Endocrinology 2007; 148: 10.1210/en.2007-0585. 10.1210/en.2007-0585 17690167

[71] Wang X, DeFranco DB. Alternative effects of the ubiquitin-proteasome pathway on glucocorticoid receptor down-regulation and transactivation are mediated by CHIP, an E3 ligase. Mol Endocrinol*.* 2005; 19: 10.1210/me.2004-0383. 10.1210/me.2004-0383 15761032

[72] Wing SS, Goldberg AL. Glucocorticoids activate the ATP-ubiquitin-dependent proteolytic system in skeletal muscle during fasting. Am J Physiol- Endocrinol Metab. 1993; 264: 10.1152/ajpendo.1993.264.4.e668. 10.1152/ajpendo.1993.264.4.E668 7682781

[73] Price SR, England BK, Bailey JL, Van Vreede K, Mitch WE. Acidosis and glucocorticoids concomitantly increase ubiquitin and proteasome subunit mRNAs in rat muscle. Am J Physiol-Cell Physiol*.* 1994; 267: 10.1152/ajpcell.1994.267.4.c955. 10.1152/ajpcell.1994.267.4.C955 7943291

[74] Marinovic AC, Zheng B, Mitch WE, Price SR, Tissue-specific regulation of ubiquitin (UbC) transcription by glucocorticoids: in vivo and in vitro analyses. Am J Physiol- Renal Physiol*.* 2007; 292: 10.1152/ajprenal.00178.2006. 10.1152/ajprenal.00178.2006 16954342

[75] Princiotta MF, Finzi D, Qian SB, Gibbs J, Schuchmann S, Buttgereit F, et al. Quantitating protein synthesis, degradation, and endogenous antigen processing. Immunity. 2003; 18: 10.1016/S1074-7613(03)00051-7. 10.1016/s1074-7613(03)00051-7 12648452

[76] Cajigas IJ, Will T, Schuman EM. Protein homeostasis and synaptic plasticity. EMBO J. 2010; 29: 10.1038/emboj.2010.173. 10.1038/emboj.2010.173 PMC2924649 20717144

[77] Choi GE, Oh JY, Lee HJ, Chae CW, Kim JS, Jung YH, et al. Glucocorticoid-mediated ER-mitochondria contacts reduce AMPA receptor and mitochondria trafficking into cell terminus via microtubule destabilization. Cell Death Dis. 2018; 10.1038/s41419-018-1172-y. 10.1038/s41419-018-1172-y PMC6235892 30429451

[78] Leschik J, Lutz B, Gentile A. stress-related dysfunction of adult hippocampal neurogenesis-an attempt for understanding resilience? Int J Mol Sci. 2021; 22: 10.3390/ijms22147339. 10.3390/ijms22147339 PMC8305135 34298958

[79] Brun P, Bégou M, Andrieux A, Mouly-Badina L, Clerget M, Schweitzer A, et al. Dopaminergic transmission in STOP null mice. J Neurochem*.* 2005; 94: 10.1111/j.1471-4159.2005.03166.x. 10.1111/j.1471-4159.2005.03166.x 15953350

[80] Andrieux A, Salin PA, Vernet M, Kujala P, Baratier J, Gory-Fauré S, et al. The suppression of brain cold-stable microtubules in mice induces synaptic defects associated with neuroleptic-sensitive behavioral disorders. Genes Dev*.* 2002; 16: 10.1101/gad.223302. 10.1101/gad.223302 PMC187434 12231625

[81] Tognini P, Napoli D, Pizzorusso T. Dynamic DNA methylation in the brain: a new epigenetic mark for experience-dependent plasticity. Front Cell Neurosci. 2015; 9: 10.3389/fncel.2015.00331. 10.3389/fncel.2015.00331 PMC4548453 26379502

[82] Buonocore G, Perrone S, Gioia D, Gatti MG, Massafra C, Agosta R, et al. Nucleated red blood cell count at birth as an index of perinatal brain damage. Am J Obstet Gynecol. 1999; 181: 10.1016/S0002-9378(99)70396-0. 10.1016/s0002-9378(99)70396-0 10601935

[83] de Goede OM, Razzaghian HR, Price EM, Jones MJ, Kobor MS, Robinson WP, et al. Nucleated red blood cells impact DNA methylation and expression analyses of cord blood hematopoietic cells. Clin Epigenet. 2015; 7: 10.1186/s13148-015-0129-6. 10.1186/s13148-015-0129-6 PMC4567832 26366232

[84] Green DW, Mimouni F. Nucleated erythrocytes in healthy infants and in infants of diabetic mothers. J Pediatr*.* 1990; 116: 10.1016/S0022-3476(05)81662-2. 10.1016/s0022-3476(05)81662-2 2248625

